# Successful management of aberrant right hepatic duct during laparoscopic cholecystectomy: a rare case report

**DOI:** 10.1186/s40792-019-0632-7

**Published:** 2019-05-09

**Authors:** Keisuke Oyama, Shin Nakahira, Hisataka Ogawa, Kazuya Kato, Makoto Hasegawa, Takayuki To, Ryosuke Maki, Hoshi Himura, Hidemi Nishi, Nobuyoshi Ohhara, Jota Mikami, Yoichi Makari, Ken Nakata, Masaki Tsujie, Junya Fujita

**Affiliations:** Department of Surgery, Sakai City Medical Center, 1-1-1 Ebarajicho, Nishi-ku, Sakai City, Osaka, 593-8304 Japan

**Keywords:** Aberrant right hepatic duct, Laparoscopic cholecystectomy

## Abstract

**Background:**

Anatomic variants of the biliary tree present challenges to surgical management during laparoscopic cholecystectomy and affect perioperative outcomes. An aberrant right hepatic duct connecting into the cystic duct is a practically important variation because of the susceptibility to serious postoperative refractory bile leakage. We report a successful case of laparoscopic cholecystectomy in the aberrant right hepatic duct of a patient diagnosed with chronic cystitis.

**Case presentation:**

A 49-year-old man was referred to our department for treatment of chronic cholecystitis. Magnetic resonance cholangiopancreatography indicated that the cystic duct branched from the common bile duct and an aberrant bile duct connected to the cystic duct. Intraoperative cholangiography revealed that the bile duct was not confluent to the major right branch of the intrahepatic bile duct and drained a narrow area. Preoperative magnetic resonance cholangiopancreatography had diagnostic value. Furthermore, intraoperative cholangiography with the Critical View of Safety method was paramount to achieving safe cholecystectomy based on confirmation of the biliary anatomy and the drainage area of the aberrant right hepatic duct.

**Conclusion:**

We encountered a rare but clinically significant case of laparoscopic cholecystectomy. This case suggests that precise understanding of the anatomy and drainage area of the aberrant right hepatic duct preoperatively and intraoperatively can lead to safe cholecystectomy.

**Electronic supplementary material:**

The online version of this article (10.1186/s40792-019-0632-7) contains supplementary material, which is available to authorized users.

## Background

Laparoscopic cholecystectomy is currently regarded as the standard surgical treatment for cholecystitis and cholecystolithiasis. Bile duct injury continues to be a serious complication of laparoscopic cholecystectomy [1] and occasionally stems from the presence of unrecognized variants of the anatomical biliary tree [2, 3]. Anatomical variants of the biliary tree include aberrant right hepatic duct (ARHD), which has an incidence of approximately 5% (1.02–35%) [4]. An ARHD drains primarily into the common hepatic duct, common bile duct, or left hepatic duct. Notably, an ARHD rarely flows into the cystic duct, and this anatomical variant occasionally accounts for an injured ARHD during surgery [5]. A rare occurrence of ARHD draining into the cystic duct requires meticulous care during cholecystectomy, as injury can prompt postoperative bile leakage and postsurgical complications. The Critical View of Safety (CVS) technique first introduced by Strasberg is based on the precise anatomical assessment and identification of biliary tree variants [1, 5] and can prevent accidental biliary and vascular injuries due to uncommon anatomical variations [1, 6].

Magnetic resonance cholangiopancreatography (MRCP) was recently reported to be an optimal imaging modality that can provide biliary tract information and accurately distinguish the presence of biliary tree variants [7]. The case presented here reports the successful application of laparoscopic cholecystectomy supported by preoperative MRCP in the management of an ARHD draining into the cystic duct of a patient with chronic cholecystitis.

### Case presentation

**Fig. 1:**
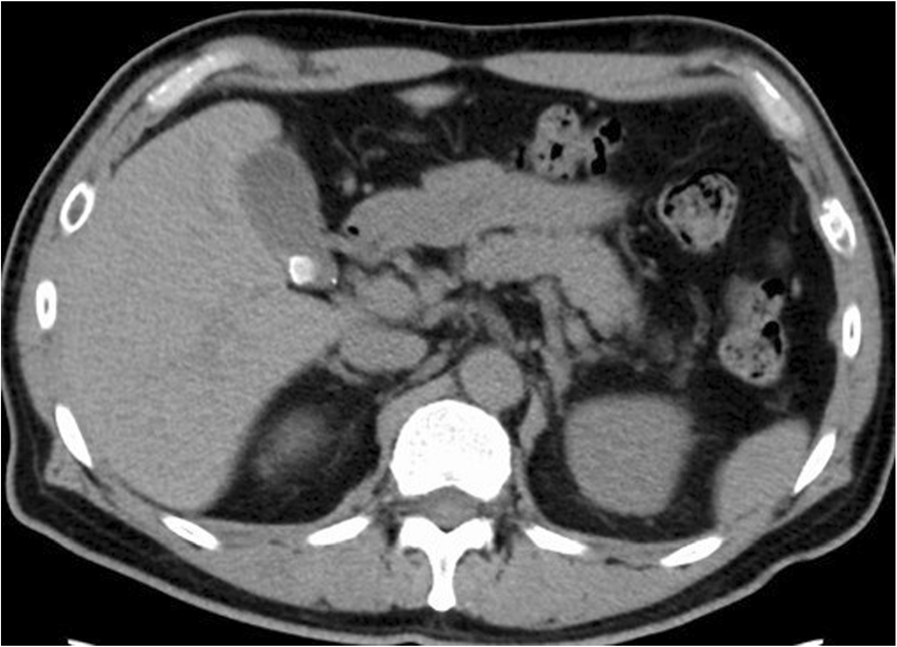
Computed tomography showing calculous cholecystitis (axial sections)

**Fig. 2:**
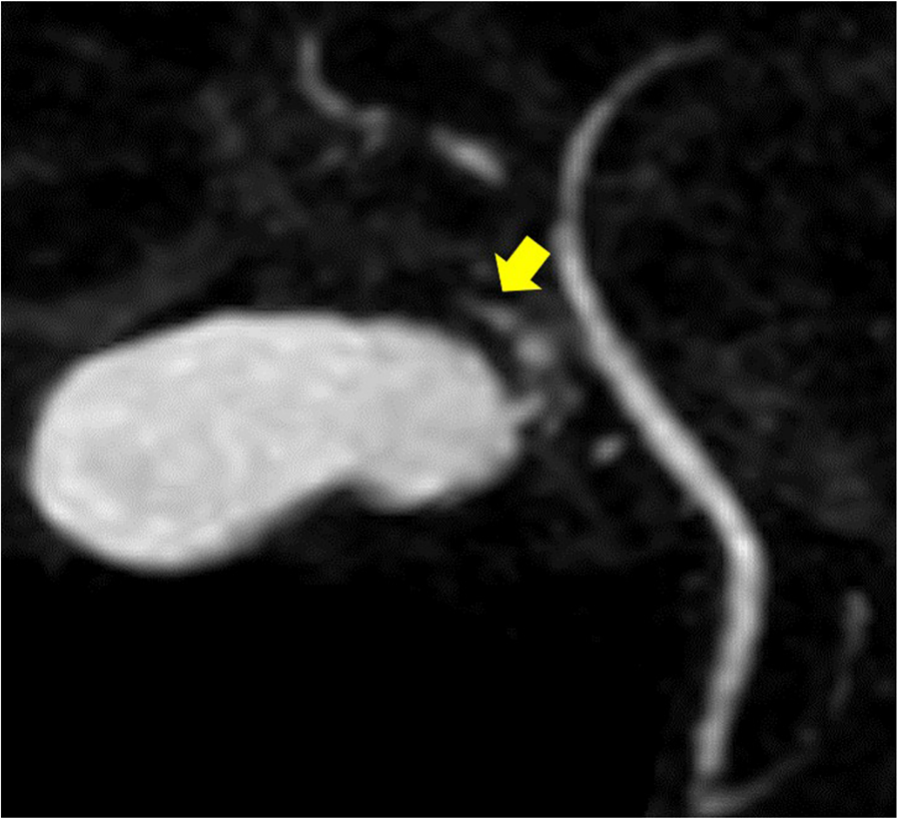
Magnetic resonance cholangiography showing the cystic duct branching from the common bile duct and an aberrant right hepatic duct (arrow) connecting to the cystic duct

A 49-year-old male with a history of cholelithiasis presenting with right hypochondoralgia with a positive Murphy’s sign was referred to our department for surgical treatment. Computed tomography (CT) without contrast media revealed a gallstone in the thickened gallbladder wall with a slight increase in the CT value due to surrounding panniculitis (Fig. 1). MRCP revealed that the cystic duct branched from the common bile duct and an aberrant bile duct connected to the cystic duct (Fig. 2, yellow arrow). The link between the aberrant bile duct and major intrahepatic biliary system was not visually apparent by MRCP. The patient was diagnosed with chronic calculous cholecystitis with aberrant bile duct flow into the cystic duct. During laparoscopic cholecystectomy, four ports were placed: a 12-mm camera port in the umbilical area by open method, 12-mm port in the epigastric area, 5-mm port in the right subcostal area, and a 5-mm port at the right lateral abdomen. Due to inflammatory fibrotic adhesion in Calot’s triangle, the ARHD was excessively exposed. A fundus-first technique was performed to confirm the ARHD after exposure of the inner layer of the subserosal layer at the dorsal and ventral side of Calot’s triangle. ARHD drainage into the cystic duct was confirmed. Preoperative MRCP suggested it was not necessary to preserve the ARHD with the extreme narrow drainage area as it seemed to be closed at the hepatic side without communicating with the major right branch of the intrahepatic bile duct.

**Fig. 3:**
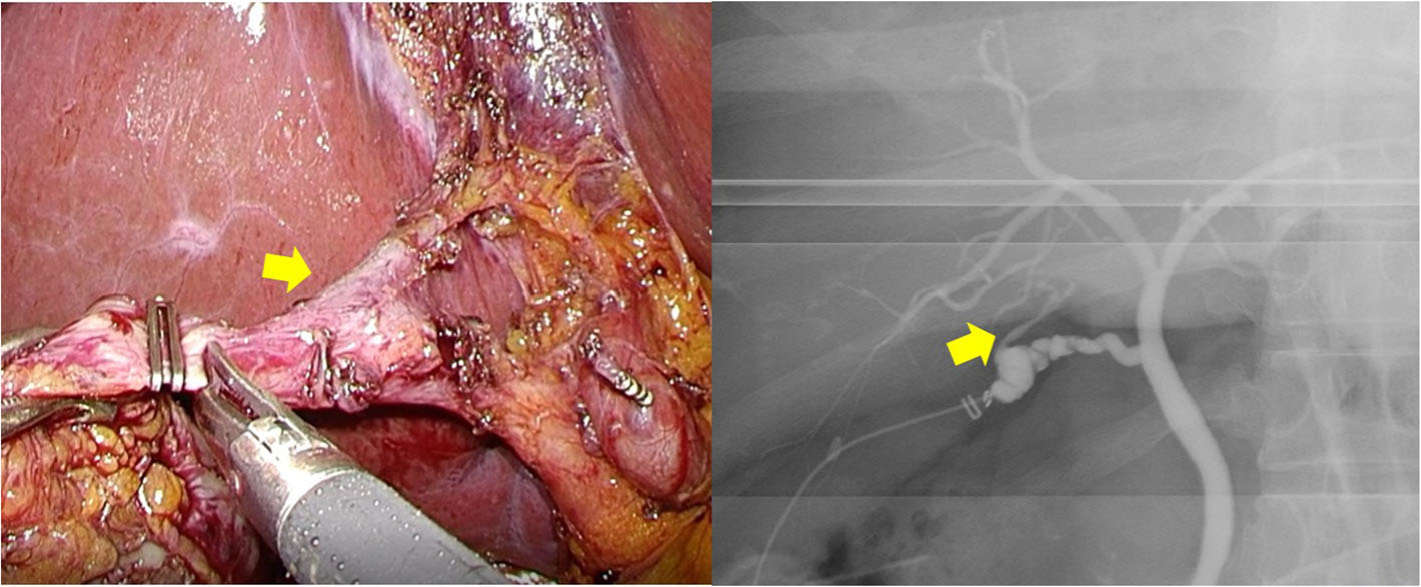
Laparoscopic exploration showing a connection between the cystic duct and aberrant right hepatic duct (arrow) ending in the liver bed. Intraoperative cholangiography showing that the aberrant right hepatic duct (arrow) was neither confluent to the major hepatic duct nor responsible for draining any liver segments

**Fig. 4:**
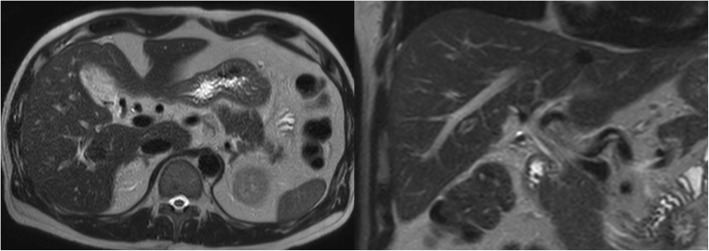
Follow-up MRCP after the operation showing no dilated bile ducts in the liver


Intraoperative cholangiography from the cystic duct in the periphery (Fig. 3) revealed that the ARHD was not confluent with the major right branch of the intrahepatic bile duct and drained a narrow area, so we removed the excessively exposed ARHD. Removal and ligation of the ARHD on the hepatic side and cystic duct was performed by clipping (AESCULAP DS Titanium Ligation Clips, B Braun brand, Tokyo, Japan). An absence of bile leakage precluded any placement of drainage. The surgical movie is available online only (Additional file 1: Video). The postoperative course was uneventful, and the patient was discharged on the third postoperative day. Follow-up MRCP showed no dilated bile duct in the liver 1 month after surgery (Fig. 4). The resected specimen was diagnosed as chronic cholecystitis. Laboratory analysis showed no abnormal increase in AST, ALT, ALP, or bilirubin 1 month after surgery (data not shown), and no abnormal symptoms 3 months after surgery. Then, his family doctor is currently following up him.

**Additional file 1:** Video Summary video of the surgery (MP4 17983 kb)


## Discussion

This case supports the importance of a meticulous understanding of biliary variations during surgery. Here, the utilization of MRCP enabled us to accurately diagnose ARHD drainage into the cystic duct before surgery. In this case, employing the CVS procedure together with the fundus-first technique provided a safe and optimal view of the confluence between the cystic duct and ARHD ending in the liver bed. Furthermore, we performed intraoperative cholangiography to judge whether the ARHD should be preserved depending on the drainage area, as it is difficult to determine an accurate corresponding drainage region. Hisatsugu et al. classified ARHD depending on the site where the ARHD and cystic duct join. According to their classification, this present case fits into the rare type V (ARHD inflows into cystic duct) with a high risk of intraoperative bile duct injury [8]. Kurata et al. also reported ARHD in 40 of 506 laparoscopic cholecystectomies in reference to Hisatsugu’s classification, and only one case (0.2%) was type V [9]. Inappropriate removal of the ARHD is reported to be associated with recurrent obstructive cholangitis, and possibly subsequent intrahepatic lithiasis, liver atrophy, and cholangiocarcinoma [4, 10]. On the other hand, preservation of the ARHD depends on the size. Longmire et al. described ligation of bile ducts smaller than 1 emm, but those larger than 2 mm or that drain one or more liver segments require reconstruction [11]. Furthermore, excessive exposure around the bile duct can induce delayed ischemic change, leading to stenosis or bile leakage [12]. In this case, intraoperative cholangiography confirmed the existence of ARHD connecting with the cystic duct and established that it was not responsible for draining any hepatic segments. From these findings, we considered that an excessively exposed ARHD should be removed to prevent delayed serious complications, and it was unnecessary to reconstruct the ARHD because of its small drainage area. Postoperative MRCP showed no dilated intrahepatic duct, which is consistent with the intraoperative judgement. Although we do not have any specific follow-up plan for a case in which the ARHD is removed, we monitored this patient and ended follow-up after no abnormal findings in the lab investigation and MRCP 3 months after surgery.

## Conclusion

We present a rare but clinically significant case of ARHD during laparoscopic cholecystectomy for the treatment of chronic calculous cholecystitis. The comprehensive combination of preoperative MRCP and intraoperative cholangiography followed by the CVS method is an effective approach for the successful management of chronic cholecystitis in patients with biliary variants.

## Abbreviations

ARHD: Aberrant right hepatic duct
CT: Computed tomography
CVS: The Critical View of Safety
MRCP: Magnetic resonance cholangiopancreatography
